# Chemical Composition and Antioxidant Activity of Steam-Distilled Essential Oil and Glycosidically Bound Volatiles from *Maclura Tricuspidata* Fruit

**DOI:** 10.3390/foods8120659

**Published:** 2019-12-09

**Authors:** Gyung-Rim Yong, Yoseph Asmelash Gebru, Dae-Woon Kim, Da-Ham Kim, Hyun-Ah Han, Young-Hoi Kim, Myung-Kon Kim

**Affiliations:** Department of Food Science and Technology, Chonbuk National University, Jeonju 54896, Jeonbuk, Korea; rudfla1226@naver.com (G.-R.Y.); holden623@naver.com (Y.A.G.); eodns3344@gmail.com (D.-W.K.); dadaham@naver.com (D.-H.K.); hha208@Korea.kr (H.-A.H.); yhoi1307@hanmail.net (Y.-H.K.)

**Keywords:** *Maclura triscuspidata* fruit, essential oil, glycosidically bound volatiles, gas chromatography-mass spectroscopy (GC–MS), chemical composition, antioxidant activity

## Abstract

Essential oil obtained from *Maclura triscuspidata* fruit has been reported to have functional properties. This study aimed at determining chemical compositions and antioxidant activities of steam-distilled essential oil (SDEO) and glycosidically bound aglycone fraction (GBAF) isolated from fully ripe *M. triscuspidata* fruit. SDEO was isolated by simultaneous steam distillation and extraction (SDE). GBAF was prepared by Amberlite XAD-2 adsorption of methanol extract, followed by methanol elution and enzymatic hydrolysis. Both fractions were analyzed by gas chromatography–mass spectrometry (GC–MS). A total of 76 constituents were identified from both oils. Apart from fatty acids and their esters, the SDEO contained *p*-cresol in the highest concentration (383.5 ± 17.7), followed by δ-cadinene (147.7 ± 7.7), β-caryophyllene (145.7 ± 10.5), β-ionone (141.0 ± 4.5), *n*-nonanal (140.3 ± 20.5), theaspirane A (121.3 ± 4.5) and theaspirane B (99.67 ± 9.05 µg/g). Thirteen carotenoid-derived compounds identified in the SDEO are being isolated from *M. triscuspidata* fruit for the first time. Out of the 22 components identified in GBAF, 14 were present only in the glycosidically bound volatiles. Antioxidant activity of the GBAF was higher than that of SDEO. These results suggest that glycosidically bound volatiles of *M. triscuspidata* fruit have a good potential as natural antioxidants.

## 1. Introduction

Plant-derived essential oils are complex mixtures of volatile and semi-volatile organic compounds characterized by diverse odors and chemical compositions depending on their origins. They are traditionally obtained from various plant tissues including fruits, seed, leaves, flowers, roots, woods and barks by means of hydrodistillation, steam distillation, solvent extraction or cold pressing [1,2]. Due to their organoleptic and biological properties, essential oils have been used as flavoring agents and natural preservatives in foods since ancient times [3]. More recently, essential oils and some of their isolated components are increasingly being used in various commercial products such as foods, cosmetics, perfumes, household cleaning products and hygiene products, and medicinal applications [2]. These compounds have been reported to have various biological activities including antimicrobial, antioxidant, antiviral, antiplatelet, antithrombotic, antiallergic, anti-inflammatory, antimutagenic, and anticarcinogenic properties [4,5,6]. 

Lipid oxidation causes serious problems in foods by producing unpleasant flavors, discoloration, decreasing nutritional quality and safety of foods through due to production of secondary oxidation products that have harmful effects on human health [7]. The use of essential oils as natural antioxidants is a field of growing interest because of the fact that synthetic antioxidants such as butylated hydroxyanisole (BHA) and butylated hydroxyltoluene (BHT) have been suspected of causing liver damage and carcinogenesis when used at high levels in laboratory animals [8,9,10,11]. For this reason, their use in the food industry has recently declined owing to safety concerns and consumer demand for natural products. 

*Maclura tricuspidata* (Carr.) Bur. (formerly known as *Cudrania tricuspidata*) which belongs to the Moraceae family is a thorny tree native to East Asia including China, Japan and Korea. The leaves, root, stem and fruit of this plant have been used in traditional herbal medicines to treat jaundice, hepatitis, neuritis and inflammation in Korea [12]. Several beneficial effects of *M. tricuspidata* extracts have been reported including anticancer [13,14], anti-inflammatory [15], antioxidant [16,17], and antidiabetes effects [18]. Various bioactive compounds such as prenylated xanthones, phenolic acids and flavonoids have already been identified from its leaves, root, stem and fruit [19,20,21]. 

The ripe fruits of *Maclura tricuspidata* which have a bright red color are edible with a floral aroma and sweet taste. They have traditionally been used to prepare fresh juice, jam, wine, vinegar and fermented alcoholic beverages in Korea. Previous studies have reported that the extracts and components of *M. tricuspidata* fruits have strong antioxidant and free radical-scavenging activities in an in vitro system [22,23]. The antioxidant activity of *M. tricuspidata* fruit extract is associated with the presence of phenolic compounds such as flavonoids and phenolic acids [17,24]. We have recently identified 18 polyphenolic compounds among which five parishin derivatives (gastrodin, parishin A, B, C, E) identified for the first time in the fruit and confirmed their anti-oxidant potentials [25]. Essential oil obtained from the fruit by microwave-assisted hydrodistillation has also been reported to have antioxidant activity through 2,2-diphenyl-1-picrylhydrazyl (DPPH), nitric oxide, hydroxy and superoxide radical scavenging activities [26]. Recently, Bajpai and colleaques [26] identified 29 compounds as major constituents in the essential oil isolated from *M. tricuspidata* fruit. Although the chemical compositions and their antioxidant activities of essential oils from the stem and root of *M. tricuspidata* were elucidated [26,27], the information on the chemical composition and antioxidant activity of the essential oil of *M. tricuspidata* fruit is still very poor. Furthermore, it is known that some volatile compounds in plants are present either in a free form and glycosidically bound forms to sugar moiety [28,29]. In some plants, glycosidically bound volatiles have shown a more potent antioxidant activity than essential oils [30,31]. Nevertheless, little is known about chemical constituents and their antioxidant potentials of glycosidically bound aglycones in *M*. *tricuspidata* fruit. Therefore, the objective of this study was to elucidate the chemical composition of steam-distilled essential oils (SDEO), aglycone fraction and major compounds of aglycone fraction liberated from glycosidically bound volatiles (GBAF) in *M. tricuspidata* fruit and their antioxidant potentials.

## 2. Materials and Methods 

### 2.1. Reagents

*n*-Decanol, *n*-decyl-β-d-glucopyranoside, Amberlite XAD-2 polymeric resin (20–60 mesh), butylated hydroxyanisole (BHA), butylated hydroxy toluene (BHT), ascorbic acid, 2,2-diphenyl-1-picrylhydrazyl (DPPH), 2,2′-azino-bis(3-ethylbenzothiazoline-6-sulfonic acid) diammonium salt (ABTS), 2,4,6-tri(2-pyridyl)-*s*-triazine (TPTZ) and saturated *n*-alkanes mixture (C_7_–C_30_), were purchased from Sigma-Aldrich Corp. (St. Louis, MO, USA). Authentic volatile chemicals were purchased from commercial sources (Sigma-Aldrich and Wako Pure Chemical Industries, Ltd., Osaka, Japan). The other reagents used were of analytical grade and were purchased from commercial sources. 

### 2.2. Plant Materials

*M. tricuspidata* fruits were collected in late October 2017 at a fully mature stage from plants cultivated in a farm located in Milyang district, Gyeongsangnam-do, Republic of Korea. A voucher specimen has been deposited at the Herbarium of Department of Food Science and Technology, College of Agricultural Life Science, Chonbuk National University. The fruit was freeze-dried for 4 day. The samples were powdered and stored in a freezer (−20 °C) until use.

### 2.3. Isolation of Steam-Distilled Essential Oil

A powdered sample (100 g) and distilled water (2 L) were placed in a 3 L round flask. The essential oil was isolated by means of simultaneous steam distillation and extraction at atmospheric pressure in a modified Likens–Nickerson type apparatus using *n*-pentane-diethyl ether (1:1) containing *n*-decanol (950 μg) as an internal standard for 2 h [32]. After the isolated oil was dried over anhydrous sodium sulfate for 12 h, the solvent was concentrated to a volume of 0.5 mL using a Vigreaux column at 40 °C and thereafter was evaporated off under a stream of nitrogen. The resulting residue was redissolved in 1 mL of *n*-pentane-diethyl ether (1:1) and subjected to gas chromatography (GC) and GC–mass spectrometry (GC–MS) analysis.

### 2.4. Isolation of Free Volatiles and Glycosidically Bound Volatiles 

The powdered sample (100 g) was homogenized with 300 mL of methanol for 1 min in a Waring blender. The homogenate was centrifuged at 4500× g for 20 min. The residue was homogenized with 300 mL of methanol followed by centrifugation as above. The supernatant was combined and the solvent was concentrated to remove methanol under reduced pressure at 40 °C. The residue was dissolved in 100 mL of distilled water and was passed through a previously preactivated (with methanol) Amberlite XAD-2 (20–60 mesh) adsorbent column (5 × 35 cm) at a flow rate of 3 mL/min according to a previously reported method [33]. After the column was washed with 1.5 L of distilled water, free volatiles (FV) and glycosidically bound volatile (GBV) fraction was isolated by sequentially eluting with each 1 L of *n*-pentane:diethyl ether (1:1) and methanol, respectively. The FV fraction was dried over anhydrous sodium sulfate for 12 h and filtered through filter paper. The filtrate was concentrated to remove solvent under reduced pressure at 40 °C. The resulting residue was redissolved in 1 mL of *n*-pentane-diethyl ether (1:1). The methanol eluate designated as GBV was concentrated under reduced pressure to dryness at 40 °C. After residue was redissolved in 50 mL of 0.1 M citrate-phosphate buffer (pH 4.8), the aqueous layer was washed triplicate with each 50 mL of *n*-pentane:diethyl ether (1:1) to remove remaining free volatiles and added *n*-decyl-β-d-glucopyranoside (1900 µg) as an internal standard. The GBF was hydrolyzed by *Aspergillus niger* cellulase (80 mg, 24 U as β-glucosidase) at 37 °C for 36 h with gentle shaking. The liberated aglycones were isolated by liquid-liquid extraction using ethyl acetate (50 mL × 3). After the liberated glycosidically bound aglycone fractioin (GBAF) was dried over anhydrous sodium sulfate for 12 h, the solvent was evaporated using rotary evaporator at 40 °C. The resulting residue was dissolved in ethyl acetate. The extracts prepared were stored at −20 °C until use. 

### 2.5. Gas Chromatography (GC) and GC–Mass Spectrometry (GC–MS) Analysis 

GC analysis was performed on a Hewlett-Packard model 6890 series gas chromatograph, with a flame ionization detector (FID), a split ratio of 1:30 using Agilent J&W DB-5MS fused silica capillary column (30 m × 0.32 mm, i.d., 0.25 μm film thickness, Santa Clara, CA, USA) and Agilent J&W Supelcowax 10 fused silica capillary column (30 m × 0.32 mm, i.d., 0.25 μm film thickness). The column temperatures were programmed from 50 °C to 230 °C at 2 °C/min and then kept constant at 230 °C for 20 min. The injector and detector temperatures were 250 °C, respectively. The carrier gas was nitrogen, at a flow rate of 1.0 mL/min. Peak areas were measured by electronic integration and the concentrations of volatile compounds were expressed as *n*-decanol equivalent (assuming response factor of all analytes was 1.0). The concentrations are to be considered only relative values as recovery after extraction and calibration factors related to the standard were not determined [34,35].

The GC–MS analysis was performed on an Agilent Technologies 7890A GC and 5975C mass selective detector operating in the EI mode at 70 eV, fitted with a DB-5MS fused silica capillary column (30 m × 0.25, i.d., 0.25 μm film thickness) and Supelcowax 10 fused silica capillary column (30 m × 0.32 mm, i.d., 0.25 μm film thickness), respectively. Both column temperatures were programmed from 50 °C to 230 °C at 2 °C per minute and then kept constant at 230 °C for 20 min. The injector and ion source temperatures were 250 °C. The carrier gas was helium at a flow rate of 1.0 mL/min. Identification of the compounds was achieved by comparing their retention times with those of authentic standards and mass spectral data in Wiley7n,1 database (Hewlett-Packard, Palo Alto, CA, USA), and NIST (National Institute of Standards and Technology, USDA) Webbook, and reported retention indices in the literatures [36]. Retention indices of each compound was calculated by a homologous series of saturated *n*-alkanes (C_7_–C_30_) (concentration of 1000 µg/mL in *n*-hexane) under the same conditions [37]. All compounds identified based on comparisons of only mass spectral data were listed as tentatively identified. 

### 2.6. Determination of Total Phenolic Content

Total phenol content of the sample was measured according to the method described by Chandra et al. [38] with some modifications. Briefly, 20 μL of each fraction (at concentration of 1000 μg/1 mL methanol) was mixed with 50% Folin–Ciocalteu phenol reagent (20 μL) in 96-well plates. After 5 min, 1 N sodium carbonate solution (20 μL) was added to the mixture and distilled water was added to adjust the final volume to 200 μL. After incubation at room temperature (RT) in the dark for 30 min, the absorbance of test sample against a blank was measured at 725 nm using a VersaMax enzyme-linked immunosorbent assay (ELISA) microplate reader (Molecular Devices, LLC, San Jose, CA, USA). Total phenol content was calculated based on a calibration curve of gallic acid. The results were expressed as mg gallic acid equivalent (mg GAE)/g.

### 2.7. Antioxidant Activity 

#### 2.7.1. Preparation of Sample

The solvent in the test samples (SDEO, FV, GBV and GBAF) were removed under a nitrogen stream. The resulting residues were dissolved in *n*-pentane:diethyl ether (1:1). BHA, BHT and ascorbic acid all diluted to a concentration of 1000 μg per mL in methanol were used as positive controls for the antioxidant activity assays.

#### 2.7.2. DPPH (2,2-Diphenyl-1-Picrylhydrazyl) Free Radical-Scavenging Activity

DPPH radical scavenging activity was determined according to the method described by Thaipong et al. [39] with some modifications. For calculation of effective concentration EC_50_ value, a stock solution of DPPH was freshly prepared by dissolving 240 mg DPPH in methanol (1000 mL) and the working solution was prepared by diluting stock solution with methanol to obtain an absorbance of 1.1 ± 0.02 units at 517 nm using an ultraviolet–visible (UV–vis) spectrophotometer (Shimadzu UV-1601, Osaka, Japan). 100 μL of the samples (SDEO, FV and TBAF) and chemicals were allowed to react with 0.1M Tris-HCl buffer (900 μL) and 500 μM DPPH solution (1000 μL) for 20 min at RT in the dark. Then absorbance was taken at 517 nm using UV–vis spectrophotometer. The EC_50_ (μg/mL) were calculated from the regression curves using six different concentrations (10–100 μg/mL) of samples and chemicals. The results were expressed as EC_50_ value (μg/mL). As a blank, the test was repeated using buffer instead of samples, and the DPPH radical-scavenging activity of the extracts was calculated against a blank as follows:

DPPH radical-scavenging activity (%) = (1 − A_0_/A_1_) × 100

where A_0_ and A_1_ are absorbance values of the test sample and control, respectively.

#### 2.7.3. ABTS (2,2′-Azino-Bis(3-Ethylbenzothiazoline-6-Sulfonic Acid)) Free Radical-Scavenging Activity

ABTS free radical scavenging activity was determined by the methods of Thaipong et al. [39] with some modifications. Briefly, a mixture of ABTS (7.4 mM) solution and potassium persulfate (2.6 mM) solution in 1:1 ratio was kept at room temperature for 12 h under dark condition to form ABTS cation. The solution was diluted by adding methanol to obtain an absorbance of 1.1 ± 0.02 at 734 nm. All the required solutions were freshly prepared for each assay. 100 μL of the samples and chemicals were added to 1400 μL of the diluted ABTS solution and the mixture was incubated at room temperature for 2 h in a dark. After the reaction, its absorbance was measured at wavelength of 734 nm. The results were expressed as RC_50_ value (μg/mL), and also ABTS radical scavenging activity (%) was calculated with the following equation:

ABTS radical scavenging activity (%) = (1 − A_0_/A_1_) × 100

where A_0_ and A_1_ are absorbance values of the test sample and control, respectively.

#### 2.7.4. Ferric-Reducing Antioxidant Power (FRAP) 

Ferric-reducing power was determined using FRAP assay [40] with some modification. The FRAP reagent was prepared by mixing 10 volume of 300 mM acetate buffer (pH 3.6) with 1 volume of 10 mM TPTZ solution in 40 mM HCl and 1 volume of 20 mM ferric chloride solution. Sample extract (75 μL) was added to 1425 μL of FRAP reagent. The reaction mixture was then incubated at RT for 30 min in a dark. The reducing power was expressed as absorbance at 593 nm and RC_50_ values (μg/mL) of FRAP were calculated from the regression lines using six different concentrations (10–100 μg/mL) in triplicate. 

### 2.8. Statistical Analysis 

All experiments were conducted in triplicate unless otherwise indicated and the results were expressed as mean ± standard deviation (SD). The statistical analysis was conducted with SPSS (ver. 10.1) for Windows and a one-way analysis of variance (ANOVA). Duncan’s multiple range tests were carried out to test any significant differences among various fruit maturity stages. Values with *p* < 0.05 were considered as significantly different

## 3. Results and Discussion 

### 3.1. Chemical Composition of the Steam-Distilled Essential Oil (SDEO) Fraction 

**Figure 1:**
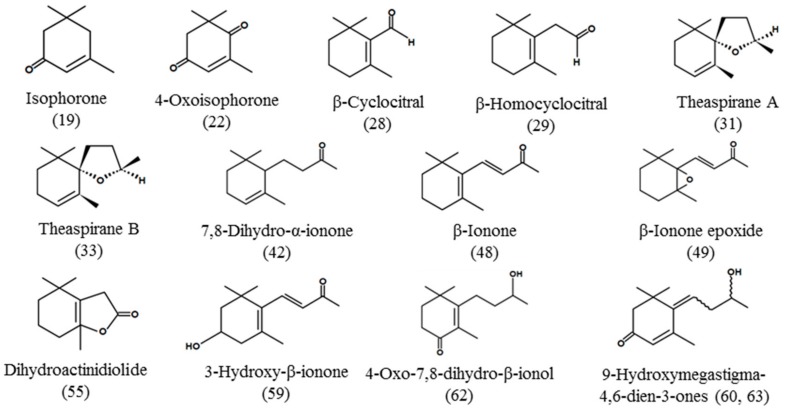
Chemical structures of carotenoid-derived compounds identified in steam-distilled essential oil (SDEO) and glycosidically bound aglycone fraction (GBAF) isolated from *M. tricuspidata* fruit. Numbers in brackets indicate peak numbers as listed in Table 1.

The yields of total SDEO and GBAF from *M. tricuspidata* fruit were 0.03 ± 0.01% and 0.37 ± 0.03%, respectively. Table 1 shows the volatile compounds identified in the SDEO and GBAF isolated from *M*. *tricuspidata* fruit along with their amounts and retention indices on DB-5MS (non-polar) and DB-WAX (polar) column. A total of 55 compounds including 17 tentatively identified compounds were identified in SDEO. The compounds that were found by only DB-5MS column but not by DB-WAX column were considered as tentatively identified. The compounds were 4 alcohols, 14 aldehyde and ketones, 7 terpenoids, 13 carotenoid-derived compounds, 6 aromatic and phenolic compounds, 11 acids and 3 miscellaneous. With the exception of aliphatic acids and their esters such as palmitic acid, linoleic acid, ethyl palmitate and linoleic acid, compounds with the highest concentration in the SDEO were *p*-cresol (393.50 ± 17.70), followed by δ-cadinene (147.67 ± 7.50), β-caryophyllene (145.67 ± 10.50), β-ionone (141.00 ± 4.40) and *n*-nonanal (140.33 ± 20.50 µg/g). In particular, 10 kinds of carotenoid-derived compounds were identified in the SDEO. These compounds have been found in various plants and are known to play an important role as characteristic aroma compounds of leaves, flowers or fruits of some plants [28,41,42]. Especially, theaspirane A and theaspirane B are present in green tea, black tea, grape and corn [43], and are believed to contribute to the unique aroma of *M. tricuspidata* fruit. Their chemical structures are presented in Figure 1. 

In this study, the norisoprenoid compounds, 7,8-dihydro-α-ionone, 3-hydroxy-β-ionone, 4-oxo-7,8-dihydro-β-ionol and 9-hydroxymegastigma-4,6-dien-3-one (two isomers) were not detected in fractions separated by the steam-distillation and extraction (SDE) method but in the glycosidicaly bound volatiles fraction (GBAF). These results suggest that most of the norisoprenoid compounds detected in the fruit are present in the form of glycosidic form rather than existing in free form in the maturing fruit or being formed in the process of preserving the fruits after harvesting [44]. These compounds can be derived from carotenoids by the action of related enzymes or chemical oxidation during processing or storage of *M. tricuspidata* fruit. It is considered that the carotenoid is decomposed in the process of separating volatile components by steam distillation. In particular, 3-hydroxy-β-ionone, 3,4-dihydro-α-ionone and two quantitatively detected 9-hydroxymegastigma-4,6-dien-3-one are present in glycosidic form in some plants [45,46]. In our previous study that analyzed phenolic compounds in the methanol extract of a fully matured fruit of the plant, we isolated a number of phenolic compounds including quercetin and parishin derivatives [25]. In this study, only 4-Hydroxybenzyl alcohol was able to be detected at a significant concentration suggesting most of the other phenolic compounds must have been degraded during the steam-distillation process.

To the best of our knowledge, 13 carotenoid-derived compounds (isophorone, 4-oxoisophorone, theaspiranes A, theaspiranes B, 7,8-dihydro-α-ionone, β-ionone, β-ionone epoxide, dihydroactinidiolide, 3-hydroxy-β-ionone, β-cyclocitral, β-homocyclocitral and two 9-hydroxymegastigma-4,6-dien-3-one isomers) are being identified for the first time from *M. tricuspidata* fruit oil. These compounds are related to carotenoids [44]. *M. tricuspidata* fruit contains several carotenoids including α-carotene, β-carotene, zeaxanthin, ruboxanthin, and lutein [47]. As described in the introduction section above, Bajpai and colleagues have previously identified 29 compounds with 1,1-difluoro-4-vinylspiropentane, scyllitol, 1-phenyl-1-cyclohexylethane, diethyl phthalate and 4,4-diphenyl-5-methyl-2-cyclohexenone as major constituents in the essential oil obtained from *M. tricuspidata* fruit by microwave-assisted extraction [26]. However, most of these compounds were not detected in this study. We believe that the difference in detected components is caused by the difference in extraction method and plant samples. In the present study, we used a fresh fruit instead of a dried one.

### 3.2. Chemical Composition of Glycosidically Bound Aglycone Fraction (GBAF) 

It is well established that volatile components in plants and foods are present in free form while some components exist in glycosidically bound forms [41,48,49]. The volatile components in the form of glycoside in association with saccharides have a hydroxyl group in the molecule and are bonded in the form of a β-glycoside. These glycosides can be hydrolyzed by β-glycosidases produced by microorganisms to produce free form of volatiles [28,33,41]. The enzyme preparation used for such a purpose are enzymes with glycosidase activities such as β-d-glucosidase, α-l-arabinopyranosidase, α-l-arabinofuranosidase and α-l-rhanosidase.

In this experiment, GBV fractions were isolated from an Amberlite XAD-2 column and then *Asp. niger* cellulase was used to release aglycones from their conjugates. Compared with the gas chromatograms of the volatile components separated by the SDE method, the number of components detected in the GBV fraction (Supplementary Figure S1) was smaller. However, it can be clearly seen that the intensities of the peaks are significantly higher in the GBV fraction. These results indicate that the overall compositions of the volatile components constituting the GBV fraction are clearly different from the volatile components present in the free form. Identities of individual compounds identified in the SDEO and GBAF are presented in Table 1.

Regarding aldehydes and ketones which belong to the oxygenated compounds, 14 components were detected in the volatile components fraction separated by the SDE method while only a small amount of phenylacetaldehyde was detected in the GBAF (Table 1). These results suggest that aldehydes and ketones present in fruits are not combined with saccharides in the form of glycosides. 

**Figure 2:**
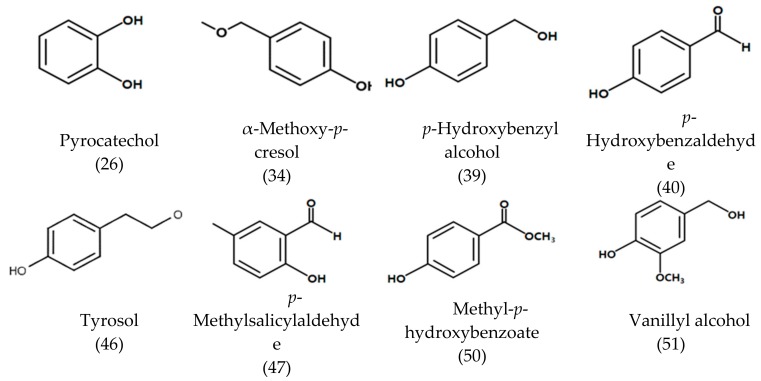

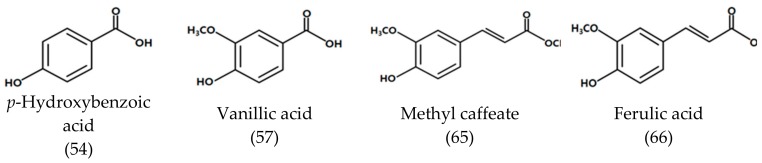
Chemical structures of aromatic and phenolic compounds identified in glycosidically bound aglycone fraction (GBAF) isolated from *M. tricuspidata* fruit. Numbers in brackets indicate peak numbers as listed in Table 1.

In the volatile fractions separated by the SDE method, few aromatic alcohol and phenolic compounds including constituents such as *p*-cresol, estragole, 2-methyl-5-(1-methylethyl) phenol, methoxy-2-methylphenol, 2,4,6-trimethylbenzaldehyde and 2-hydroxy-4-methylbenzaldehyde were detected in lower concentrations. By contrast, in the GBAF fraction, a large amount of aromatic alcohols and phenolic compounds were detected. Among them, benzyl alcohol, 2-phenylethyl alcohol, resorcinol, α-methoxy-*p*-cresol, *p*-hydroxybenzyl alcohol, *p*-hydroxybenzaldehyde, 4-methylsalicylaldehyde, methyl *p*-hydroxybenzoate, ferulic acid, methyl caffeate, pyrocatechol, *p*-hydroxyphenylethyl alcohol, vanillyl alcohol, *p*-hydroxybenzoic acid, methyl vanillate, vanillic acid, and *p*-(*p*-hydroxybenzyl) phenol were detected only in the GBAF (Table 1). The chemical structures of the phenolic compounds detected in the GBAF are shown in Figure 2. As shown in the figure, one or more hydroxyl groups are contained in the molecular structure, and thus the β-glycoside bond is hydrolyzed by treating β-glucosidase in the presence of sugar in the form of β-glycoside in the hydroxyl group. These compounds are smaller in molecular weight and simple in structure compared to other phenolic compounds, but are widely distributed in plants and are known to contribute to various physiological activities. Interesting biological activities have been reported for tyrosol, *p*-hydroxybenzyl alcohol and *p*-hydroxybenzaldehyde including anti-oxidant activities, improving functional blood flow, preventing memory deficits, and providing protective effects on the blood–brain barrier [50,51,52,53].

### 3.3. Total Phenol Contents of Fractions

**Figure 3:**
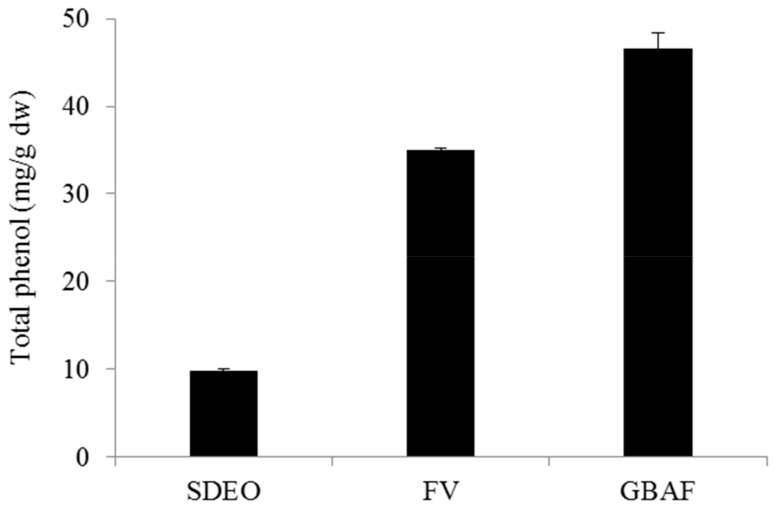
Total phenol contents of fractions isolated from *M. tricuspidata* fruit. SDEO, steam-distilled essential oil; FV, free volatile; GBAF, glycosidically bound aglycone fraction liberated from GBV by *Asp. nige*r cellulose; GBV, glycosidically bound volatile fraction.

The total phenol contents of the SDEO, FV and GBAF were also determined and comparisons of the results are presented in Figure 3. Among all, the highest total phenol content was obtained from the GBAF while the SDEO showed the lowest total phenol content (<10 mg/g dw). The total phenol content of the FV fraction was slightly lower than the GBAF while it was much higher than that of SDEO. The relatively higher total phenol contents in the GBAF and FV is due to the solvents used as the efficiency of the phenolics extraction depends on the type of the solvent. During isolation of the GBAF, extraction of the aglycones liberated by enzymatic hydrolysis employed a more polar solvent (ethyl acetate) while only *n*-pentane-diethyl ether (1:1) was used in the case of SDEO. It is well established that phenolic compounds are extracted more efficiently with polar solvents [54]. 

### 3.4. Antioxidant Activity of SDEO and GBAF

**Figure 4:**
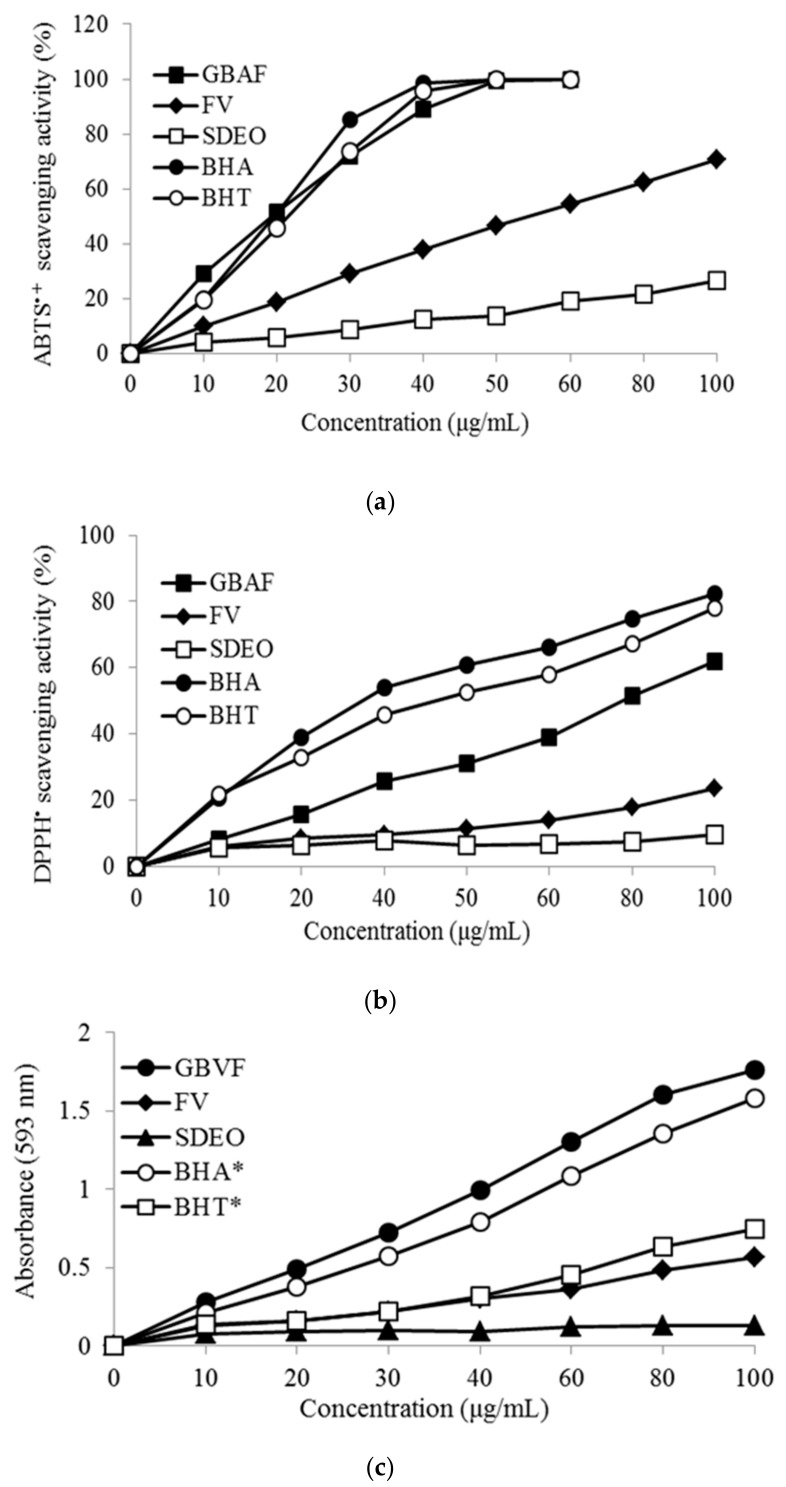
Antioxidant activities of steam-distilled essential oil (SDEO), free volatile (FV) and glycosidically bound aglycone fraction (GBAF) isolated from *M. tricuspidata* fruit. (**a**) 2,2-Diphenyl-1-Picrylhydrazyl (DPPH) free radical scavenging activity, (**b**) 2,2′-Azino-Bis(3-Ethylbenzothiazoline-6-Sulfonic Acid (ABTS) free radical scavenging activity; (**c**) Ferric reducing antioxidant power (FRAP). Samples, 1000 ug/mL; * Butylated hydroxyltoluene BHA, Butylated hydroxyanisole (BHT), 200 ug/mL.

Antioxidant activities of fruit extracts have been characterized extensively [55]. In this study, antioxidant capacities of each fraction expressed in percent of radical (DPPH and ABTS) scavenging activities and reducing power as measured by FRAP assay, and EC_50_ as compared to the positive controls BHA and BHT, are presented in Figure 4 and Table 2. In all the antioxidant property measurement methods, the GBAF showed the highest antioxidant activity while the SDEO showed the lowest. Considering the total yields of these fractions and their respective total phenol content results described above, it can be said that there is a strong positive correlation between their concentrations and their respective antioxidant activities. Maximum antioxidant activities of the GBAF were obtained in the DPPH and FRAP methods where its activity was even higher or equivalent to those of the synthetic antioxidants BHA and BHT. While the antioxidant properties of phenolic compounds are extensively demonstrated in the literature, some of the volatile compounds exclusively detected in the GBAF might also have greatly contributed to its considerable antioxidant capacity observed in this study. It should also be noticed that the volatile aroma components detected in higher concentrations in the GBAF including *p*-Hydroxybenzyl alcohol, *p*-hydroxybenzaldehyde and tyrosol are well known to have strong biological activities [56,57,58]. However, while antioxidant activity estimations based on synthetic radicals are indispensable tools, many people raise concerns about their substantiation through in vivo and clinical trials which also have more safety issues [59].

Even though antioxidant activity of the SDEO was found to be much lower than the other fractions, it is suggested that its observed antioxidant property is related to the compounds detected in it. Compounds such as palmitic acid, linoleic acid and *p*-cresol that were detected in relatively higher concentrations in the SDEO are not important antioxidants [60,61]. Generally, the antioxidant capacity of volatile compound fractions from *M. triscuspidata* fruit extracted with the SDE method and that of GBAF are attributed to the individual components identified. The antioxidant activities expressed in EC_50_ of some individual phenolic compounds evaluated in this study are also presented in Table 3. Based on these results, it can be suggested that as most potent bioactive compounds are glycosidically bound forms in *M. triscuspidata* fruit, enzymatic processing like fermentation can play an important role in enhancing its biological activities.

### 3.5. Antioxidant Activity of Individual Phenolic Compounds in GBAF

In order to evaluate the antioxidant activities of individual compounds, EC_50_ of 12 compounds identified in the GBAF was determined and the results are presented in Table 3. In all the three assay methods, methyl caffeate displayed by far the strongest antioxidant activity expressed in EC_50_. Pyrocatechol also showed the highest DPPH scavenging activity and was even higher than the synthetic antioxidants BHA and BHT. As can be seen from Table 3, several phenolic compounds including pyrocatechol, vanillyl alcohol, methyl caffeate and ferulic acid have shown antioxidant potencies higher than that of positive controls. Ferulic acid and methyl caffeate, the two compounds that showed the highest DPPH-scavenging activities in this study, have been previously reported to have antioxidant activities expressed in EC_50_ of DPPH scavenging activity of 22 and 10.64 μg/mL for ferulic acid methyl caffeate, respectively [50,62].

Therefore, it can be assumed that these compounds have greatly contributed to the overall higher antioxidant activity observed in the GBAF. As described above, only a few phenolic compounds were detected in lower concentrations in the SDEO fraction. Considering this, proper processing techniques are required before application of *M. triscuspidata* fruit for its biological activity. While processing techniques such as specific enzymatic treatments can help release some compounds, processing methods like fermentation with microorganisms may give more efficient results. A previous study has demonstrated an increase in the levels of phenolic compounds such as kaempferol and quercetin after lactobacillus-mediated fermentation of *M. triscuspidata* leaf [63].

## 4. Conclusions

This study explored the chemical compositions and antioxidant activities of steam-distilled essential oil (SDEO) and glycosidically bound aglycone fraction (GBAF) extracts from fully ripe *M. triscuspidata* fruit. Thirteen carotenoid-derived compounds are being isolated for the first time in *M. triscuspidata* fruit. These compounds have been associated with a variety of organoleptic properties in other plants. A number of bioactive compounds were exclusively identified in the GBAF. It can be suggested that the relatively higher antioxidant activity observed in this particular fraction compared to the SDEO fraction is mainly associated with these exclusive compounds. Therefore, enzymatic treatments of fruits suh as *M. triscuspidata* can significantly enhance functional properties by releasing glycosidically bound bioactive components.

## Figures and Tables

**Table 1:** Concentration of compounds identified in steam-distilled essential oil (SDEO) and glycosidically bound aglycone fraction (GBAF) isolated from *M. tricuspidata* fruit.

PeakNo	tR (min)	Compounds	RI ^1)^	RI ^2)^	Concentration (μg/100 g dw) ^3)^	
					SDEO	GBAF
Alcohols						
1	5.363	2-Methyl-1-butanol	737	1206	3.03 ± 0.25	1036.0 ± 124.6
5	7.735	*tran*s-2-Hexen-1-ol	862	1405	7.33 ± 1.53	− ^5)^
8	10.318	5-Methyl-2-furfuryl alcohol	956	− ^4)^	3.17 ± 0.76	-
20	19.585	3,4-Dimethylcyclohexanol ^6)^	1109	-	15.67 ± 2.08	-
Aldehydes and ketones						
3	6.644	Furfural	819	1459	53.67 ± 6.03	-
4	7.378	*trans*-2-Hexenal	848	1201	10.33 ± 3.06	-
2	6.284	*n*-Hexanal	804	1097	3.00 ± 0.80	-
7	8.777	2-Acetyl furan	903	1493	5.33 ± 1.53	-
9	10.513	5-Methylfufural	966	1508	4.13 ± 0.81	-
10	12.102	Benzaldehyde	971	1508	6.33 ± 1.53	-
11	12.491	6-Methyl-5-hepten-2-one	989	1326	6.33 ± 1.53	-
12	13.267	1-(2-Furanyl)-3-butanone ^6)^	1006	-	4.03 ± 0.55	-
16	15.179	Phenylacetaldehyde	1039	1629	44.33 ± 3.51	5.33 ± 1.04
18	18.958	*n*-Nonanal	1104	1388	140.3 ± 20.5	-
23	22.487	10-Undecenal ^6)^	1146	-	6.67 ± 2.52	-
24	23.306	2,4-Dimethylbenzaldehyde ^6)^	1158	1712	7.03 ± 1.55	-
44	41.734	Genanyl acetone	1451	1860	17.33 ± 2.52	-
52	44.505	2-Tridecanone	1493	-	23.67 ± 5.51	-
Terpenoids						
36	35.657	Ylangene	1356	1464	10.93 ± 3.10	-
37	36.379	α-Copaene	1368	1477	62.33 ± 51.47	-
41	39.05	β-Caryophyllene	1409	1565	145.7 ± 10.5	-
43	39.533	α-Bergamotene	1416	1575	5.67 ± 0.58	-
45	41.982	β-Humulene	1454	-	10.33 ± 2.52	-
53	45.905	δ-Cadinene	1517	1754	147.7 ± 7.5	-
58	50.101	Caryophyllene oxide	1588	1968	56.33 ± 3.51	-
Carotenoid-derived compounds						
14	15.079	2,2,6-Trimethylcyclohexanone ^6)^	1037	1300	5.57 ± 0.51	-
19	19.303	Isophorone	1119	1578	7.10 ± 1.85	-
22	21.727	4-Oxoisophorone ^6)^	1115	1674	5.33 ± 0.58	-
28	26.163	β-Cyclocitral	1214	1603	17.10 ± 1.85	-
29	28.870	β-Homocyclocitral ^6)^	1254	-	15.10 ± 0.85	-
31	31.253	Theaspirane A	1289	1482	121.3 ± 4.5	-
33	32.447	Theaspirane B	1306	1522	99.67 ± 9.02	
42	39.454	7,8-Dihydro-α-ionone ^6)^	1415	1825	-	30.33 ± 2.52
48	43.389	β-Ionone	1480	1907	141.0 ± 4.4	-
49	43.637	β-Ionone epoxide	1483	1957	92.33 ± 9.71	-
55	46.692	Dihydroactinidiolide ^6)^	1530	2291	10.67 ± 5.51	-
59	56.486	3-Hydroxy-β-ionone ^6)^	1698	2646	-	160.7 ± 30.0
60	57.969	9-Hydroxymegastigma-4,6-dien-3-one (isomer #1) ^6)^	1705	2677	-	197.67 ± 9.45
61	58.525	4-Oxo-7,8-dihydro-β-ionol	1725	2694	-	76.00 ± 11.00
63	61.311	9-Hydroxymegastigma-4,6-dien-3-one (isomer #2) ^6)^	1786	2846	-	234.3 ± 24.5
Aromatic and phenolic compounds						
15	15.292	Benzyl alcohol	1040	1864	-	883.7 ± 29.8
17	18.294	*p*-Cresol	1092	2074	393.5 ± 17.7	43.00 ± 7.55
21	19.694	2-Phenylethyl alcohol	1113	1892	-	58.85 ± 4.58
26	25.427	Pyrocatechol ^7)^	1203	2646	-	20.33 ± 5.51
30	31.225	Resorcinol	1288	-	-	57.33 ± 10.50
32	31.523	Carvacrol	1293	2213	19.37 ± 3.46	-
34	34.006	α-Methoxy-*p*-cresol ^7)^	1331	2490	-	2783.0 ± 143.0
35	34.981	*p*-Vinylguaiacol	1346	2181	-	17.33 ± 3.51
25	24.925	Methyl chavicol	1171	1658	66.67 ± 9.02	-
38	37.539	2,4,6-Trihydroxybenzaldehyde	1386	-	9.33 ± 1.53	-
39	38.473	*p*-Hydroxybenzyl alcohol ^7)^	1400	2952	17.67 ± 3.06	468.1 ± 30.9
40	38.977	*p*-Hydroxybenzaldehyde ^7)^	1408	2964	-	170.0 ± 19.5
46	42.529	Tyrosol ^7)^	1463	2969	-	68.67 ± 4.51
47	43.524	*p*-Methylsalicylaldehyde ^7)^	1478	-	43.00 ± 10.82	4088.0 ± 147.8
50	44.116	Methyl *p*-hydroxybenzoate ^7)^	1487	1969	-	289.3 ± 12.5
51	44.439	Vanillyl alcohol ^7)^	1492	-	-	30.67 ± 3.27
54	46.293	*p*-Hydroxybenzoic acid ^7)^	1523	-	-	20.33 ± 4.51
56	46.955	Methyl caffeate ^7)^	1532	2593	-	31.33 ± 4.51
57	48.027	Vanillic acid ^7)^	1583	-	-	22.67 ± 4.04
65	64.199	Methyl ferulate	1844	-	-	92.00 ± 28.62
66	65.320	Ferulic acid ^7)^	1865	-	-	383.0 ± 26.6
76	79.425	*p*-(*p*-Hydroxybenzyl)phenol ^6)^	2166	-	-	133.1 ± 12.9
Aliphatic acids and esters						
62	61.180	Myristic acid	1775	2694	124.2 ± 10.3	-
64	61.871	Ethyl myristate	1798	2041	9.33 ± 1.53	-
67	65.807	Pentadecanoic acid	1875	2822	9.33 ± 2.52	-
68	68.480	Methyl palmitate	1928	2212	55.75 ± 6.23	-
69	71.900	Palmitic acid	1986	2953	813.1 ± 39.5	-
70	72.100	Ethyl palmitate	2002	2277	291.7 ± 29.0	-
71	76.479	Methyl linoleate	2120	2485	58.16 ± 8.23	-
72	76.776	Methyl linolenate	2119	2484	55.35 ± 10.53	-
73	79.580	Linoleic acid	2169	-	363.7 ± 39.0	-
74	79.897	Linolenic acid	2175	-	176.0 ± 22.5	-
75	80.583	Ethyl linolenate	2187	2585	9.33 ± 1.53	-
Miscellaneous						-
6	7.987	*p*-Xylene	836	1279	4.17 ± 0.76	-
13	14.526	2-Acetylthiazole ^6)^	1027	-	4.03 ± 0.35	-
27	25.537	2,3-Dihydrobenzofuran	1205	2381	3.77 ± 0.68	316.0 ± 29.0

^1)^ Retention indices on DB-5MS column. ^2)^ Retention indices on Suplecowax 10 column. ^3)^ Values expressed as equivalents of *n*-decanol are given as mean ± standard deviation (*n* = 3). ^4)^ Not detected or larger retention indices than 3000 in Supelcowax 10 column. ^5)^ Not detected or less than 1.0 μg/100 g. ^6)^ Tentatively identified based on mass spectral data only due to lack of authentic standard compound. ^7)^ Compounds used for antioxidant activity assays.

**Table 2:** Antioxidant activity of SDEO, FV and GBAF isolated from *M. tricuspidata* fruit.

Samples	DPPH ^1^	ABTS ^+1^	FRAP ^2^
SDEO	17,065.22 ± 146.27 ^a^	1921.81 ± 49.45 ^a^	10,638.56 ± 223.33 ^a^
FV	2507.18 ± 24.21 ^b^	660.72 ± 7.18 ^b^	1963.48 ± 10.97 ^b^
GBAF	835.33 ± 6.97 ^d^	317.09 ± 1.99 ^d^	529.6 ± 4.73 ^d^
BHA	466.79 ± 7.10 ^e^	89.15 ± 4.14 ^e^	129.46 ± 1.61 ^f^
BHT	535.75 ± 3.52 ^e^	108.62 ± 1.06 ^e^	331.26 ± 4.68 ^e^

^1^ EC_50_ (μg/mL) values were calculated from the regression lines using six different concentrations (10–100 μg/mL) in triplicate and data represent 50% scavenging activity. ^2^ Ferric-reducing antioxidant power (FRAP) were calculated from the regression lines using six different concentrations (10–100 μg/mL in triplicate and the values were presented by sample concentration at 0.5 of absorbance at 517 nm DPPH, 2,2-Diphenyl-1-Picrylhydrazyl; ABTS, 2,2′-Azino-Bis(3-Ethylbenzothiazoline-6-Sulfonic Acid; SDEO, steam-distilled essential oil; FV, free volatile; GBAF, glycosidically bound aglycone fraction liberated from glycosidically bound volatile fraction by *Asp. niger* cellulase. Different superscripts in the same column indicate significant differences (*p* < 0.05). ^+^, cation.

**Table 3:** Antioxidant activity of phenolic compounds identified in GBAF.

Compounds	EC_50_ (μg/mL)		
	DPPH ^1^	ABTS ^1^	FRAP ^2^
Pyrocatechol	9.59 ± 1.22 ^e^	67.68 ± 2.47 ^jk^	74.45 ± 2.16 ^jk^
α-Methoxy-*p*-cresol	1114.09 ± 114.45 ^d^	59.55 ± 6.46 ^jk^	3298.92 ± 126.20 ^f^
*p*-Hydroxybenzyl alcohol	3357.55 ± 134.15 ^c^	377.85 ± 4.78 ^f^	2854.37 ± 43.04 ^g^
*p*-Hydroxybenzaldehyde	1765.90 ± 364.23 ^d^	1117.70 ± 7.01 ^c^	7906.18 ± 60.96 ^c^
Tyrosol	1331.74 ± 195.63 ^d^	287.36 ± 3.70 ^g^	92.64 ± 1.97 ^jk^
*p*-Methylsalicylaldehyde	1644.14 ± 365.52 ^d^	423.69 ± 3.13 ^e^	19,365.27 ± 81.38 ^b^
Methyl *p*-hydroxybenzoate	5241.03 ± 941.54 ^b^	12,735.03 ± 47.26^a^	6789.61 ± 82.27 ^d^
Vanillyl alcohol	27.96 ± 1.65 ^e^	66.98 ± 1.99 ^jk^	5928.60 ± 90.87 ^e^
*p*-Hydroxybenzoic acid	10,906.51 ± 1103.69 ^a^	6921.86 ± 50.48 ^b^	1116.61 ± 11.69 ^h^
Vanillic acid	48.58 ± 2.50 ^e^	157.22 ± 4.83 ^h^	161.18 ± 4.25 ^jk^
Methyl caffeate	11.92 ± 0.48 ^e^	11.91 ± 1.29 ^l^	7.84 ± 0.28 ^k^
Ferulic acid	24.47 ± 2.59 ^e^	66.39 ± 2.11 ^jk^	138.98 ± 3.73 ^jk^
BHA	26.10 ± 0.42 ^e^	89.27 ± 4.01 ^ij^	129.46 ± 1.61 ^jk^
BHT	33.71 ± 1.04 ^e^	108.76 ± 3.93 ^i^	331.26 ± 4.68 ^j^

^1^ EC_50_ (μg/mL) values were calculated from the regression lines using six different concentrations (10–100 μg/mL) in triplicate and data represent 50% scavenging activity. ^2^ FRAP were calculated from the regression lines curve using six different concentrations (10–100 μg/mL) of authentic standards in triplicate and the values were presented by sample concentration at 0.5 of absorbance at 517 nm. Different superscripts in the same column indicate significant differences (*p* < 0.05).

## Supplementary Materials

The following are available online at https://www.mdpi.com/2304-8158/8/12/659/s1, Figure S1: Gas chromatograms of the volatile components detected in (A) steam distilled essential oil (SDEO); (B) glycosidically bound aglycone fraction (GBAF) isolated from Maclura triscuspidata.
