# Supplementary material for: Chemical Composition and Antioxidant Activity of Steam-Distilled Essential Oil and Glycosidically Bound Volatiles from Maclura Tricuspidata Fruit
Source: Foods. 2019 Dec 9;8(12):659. doi: 10.3390/foods8120659 (PMC6963948; doi:10.3390/foods8120659)
Supplement: Supplementary file 1 [file foods-08-00659-s001.pptx]

## Slide 1
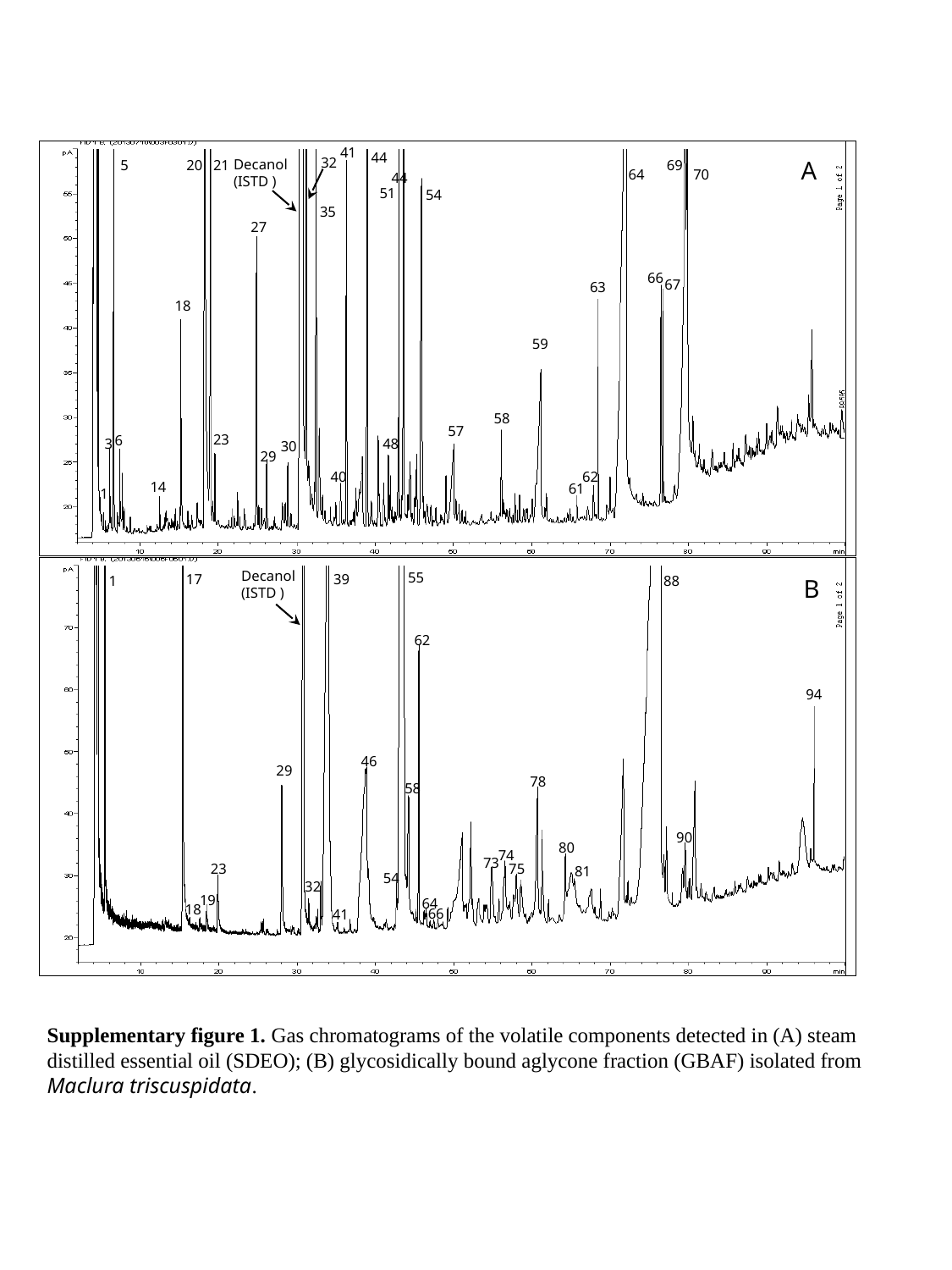

41
44
32
Decanol
(ISTD )
A
20
5
21
69
64
70
44
51
54
35
27
66
67
63
18
59
58
57
23
6
48
3
30
29
62
40
14
61
1
Decanol
(ISTD )
55
17
39
1
88
B
62
94
46
29
78
58
90
80
74
73
23
75
81
54
32
19
64
18
66
41
Supplementary figure 1. Gas chromatograms of the volatile components detected in (A) steam distilled essential oil (SDEO); (B) glycosidically bound aglycone fraction (GBAF) isolated from Maclura triscuspidata.
